# Effect of the Strategic Thinking, Problem Solving Skills, and Grit on the Disaster Triage Ability of Emergency Room Nurses

**DOI:** 10.3390/ijerph19020987

**Published:** 2022-01-16

**Authors:** Jina Yang, Kon Hee Kim

**Affiliations:** 1 Emergency Room, Myongji Hospital, Ilsan 10475, Korea; yang.jina119@gmail.com; 2 College of Nursing, Ewha Womans University, Seoul 03760, Korea

**Keywords:** thinking, problem-solving, grit, triage, nurse

## Abstract

In this descriptive study, we aimed to identify factors related to emergency room nurses’ disaster triage ability. A total of 166 nurses who worked for emergency departments of general hospitals completed a structured questionnaire consisting of the Disaster Triage Ability Scale (DTAS), the Strategic Thinking Scale (STS), the Problem-Solving Inventory (PSI), and the Original Grit Scale (Grit-O). The data were analyzed using SPSS/WIN 25.0 by means of descriptive statistics, *t*-test, one-way ANOVA, the Scheffé post hoc test, Pearson’s correlation coefficients, and stepwise multiple regression. Participants’ DTAS averaged 14.03 ± 4.28 (Range 0–20) and showed a statistically significant difference according to their experience of triage education (t = 2.26, *p* = 0.022) as a disaster triage-related attribute. There were significant correlations among DTAS and confidence in the PSI (r = 0.30, *p* < 0.001), the approach-avoidance style in the PSI (r = −0.28, *p* < 0.001), and futurism in the STS (r = 0.19, *p* = 0.019). The strongest predictor was confidence in the PSI; in addition, 14.1% of the DTAS was explained by confidence in the PSI, approach-avoidance in the PSI, and futurism in the STS. Emergency room nurses who received triage education showed a higher level of the DTAS and their DTAS could be explained by problem-solving skills and strategic thinking. Therefore, it is necessary to develop and implement triage education programs integrated with stress management to improve the approach-avoidance style to ensure better problem-solving skills and to utilize various training methods to enhance confidence to improve problem-solving skills and futurism as part of strategic thinking.

## 1. Introduction

Disasters are no longer uncommon. In the case of a disaster with multiple casualties, severity classification is essential for efficient use of limited manpower and resources [1]. Nurses are mainly responsible for the triage role in the clinical setting. Rapid information collection and accurate decision-making—that is, clinical reasoning ability—are essential for severity classification. In order to acquire this ability, continuous and systematic education is needed, and for the development and application of severity classification education programs, it is necessary to investigate the degree of nurses’ severity classification abilities and their influencing factors.

Understanding and implementing severity classification in disasters with mass-casualty incidents is vital in order to disperse any limited resources among victims with the highest possibility of survival. Recent previous mass-casualty incident studies have found that improper first aid and victim transfer distribution results in re-transfer due to inadequate severity classifications at the mass-casualty incident site [2,3]. Therefore, it is essential to perform proper severity classification in mass-casualty incident situations because it directly affects the decline of resources and the health of casualties.

The individual who performs a severity classification at the disaster site must be a competent medical professional who can identify the incident site, allocate resources, set priorities, and transfer the victims according to patient severity [4]. Emergency room triage nurses (ERTNs) are exemplar healthcare professionals who can perform such tasks [5]. Research has revealed that more than 90% of the medical professionals who perform severity classification using the Korea Triage and Acuity Scale (KTAS) are nurses. Taken together, these data suggest that ERTNs are the ideal medical professionals to make the most appropriate choices according to victim health problems and the amount of resources available in disastrous situations.

Severity classification requires strategic thinking, which involves deciding upon new and appropriate actions based on existing methods and facts learned from specific situations in order to achieve the best goal in a given situation [6]. Strategic thinking provides indisputable facts through formal analysis and refers to making a choice among different solutions that systematically minimizes the risk factors when damage occurs to an organization [7]. One study revealed that classification strategies affect severity classifications and, subsequently, the health outcomes of patients in disastrous pediatric events [8]. Therefore, strategic thinking is required in order to determine the method and range of severity classifications using the type and disaster scales and assessing the health state of casualties.

Severity classification requires an understanding of the overall situation of a disaster, resource availability, the number and condition of victims, the classification of skills based on strategic and critical thinking, and professional medical knowledge, which must be used in a timely manner. Problem-solving skills in nursing refer to the ability to resolve health issues promptly through critical thinking, based on knowledge and experience [9,10]. Problem-solving skills require self-confidence, approach-avoidance style, and control. Confidence has a positive effect on the problem-solving process and outcome. Approach-avoidance style refers to the approach style of working hard to avoid bad things, and it is similar to coping with stress. In the process of solving problems, control over one’s consciousness and behavior is required [11]. In severity classifications, problem solving skills are essential and affect accuracy through strategic and critical thinking. A previous study has shown that problem-solving skills improve the accuracy of severity classifications [12].

In a disastrous situation that is full of uncertainty, personal characteristics such as passion, persistency, and courage in spite of failure are required in those who perform severity classification. Grit refers to the characteristic of courage or determination that is required for successful goal achievement [13]. It does not mean simply passion and perseverance, but also includes the courage and persistence necessary to do the job in spite of failures without being discouraged [14]. Previous study results have shown high levels of stress in ERTNs who performed severity classification in mass-casualty incident simulation training. Therefore, grit is necessary to perform severity classification accurately in a stressful disaster situation.

Disaster triage ability (DTA) includes making the most appropriate choice for the best outcome according to the health issues of the victims and limited resources, and demonstrating courage, passion, and persistence in order to practice successful leadership. Furthermore, severity classification results influence the disaster cycle in relation to recovery; therefore, there is a need for ERTNs to have DTA that is based on specialized knowledge and skills in emergency situations. In this study, we investigated strategic thinking, problem-solving skills, grit, and DTA in ERTNs. Additionally, we examined these factors’ relationships and influences on DTA, and aimed to provide a data-based framework for the development of severity classification training.

## 2. Materials and Methods

### 2.1. Design

This study was a descriptive study that sought to demonstrate the effects of strategic thinking, problem solving skills, and grit on DTA.

### 2.2. Participants

The participants were chosen from ERTNs in general hospitals located in metropolitan areas who had a minimum of 1 year of experience, who could efficiently and promptly classify patients based on emergency medical knowledge [15,16]. The sample size was calculated using G*Power 3.1.9.4 under the conditions of a significance level (α) of 0.05 and power (1 − β) of 0.80 for multiple regression analysis [17]. According to the Cohen criterion, the medium effect size (f^2^) and predictors were set to 0.15 and 22, respectively, to calculate the number of samples required, which was 163. Considering the dropout rate, 195 questionnaires were distributed and 86.6% (169) copies were collected [18]. Three insufficient responses were excluded. Consequently, 166 responses from ERTNs were used in this study.

### 2.3. Measurements

#### 2.3.1. General and Severity Classification-Related Characteristics

A questionnaire assessing the general (gender, year of birth, education level, clinical experience, and ER experience) and severity-classification-related (disaster education participation, disaster triage education participation, disaster triage experience, KTAS qualification) characteristics were used.

#### 2.3.2. Strategic Thinking

The Strategic Thinking Scale (STS), which was developed by Salavati et al. [7], was translated and used in this study, with the authors’ permission. Translation was conducted according to the WHO translation guidelines [19], in the following sequence: forward translation; expert panel back translation; pre-testing and cognitive interviewing; and the final version. Briefly, a bilingual United States registered nurse with over ten years of ER experience conducted the forward translation. The tool translated into Korean was then back-translated by a professional translator with a PhD in translation (Korean-English). To determine culturally different expressions and the degree of correspondence between the original tool and the translated version, a panel supervised the document. This panel included a nurse with at least ten years of ER clinical experience, a professional translator with a PhD in Korean-English translation, and an English native speaker. Their opinion indicated that there were no cultural differences; however, some word changes were required and the tool was modified accordingly. Next, pre-testing was conducted on five nurses with at least one year of ER experience. The final tool was approved through cognitive interviewing, which confirmed that the content was appropriate and did not contain any issues in understanding and responding. This five-point Likert scale had four sub-areas with 26 items: system thinking, conceptual thinking, futurism, and intelligent opportunism. Higher scores indicated a higher strategic thinking ability. Cronbach’s α was 0.85 when the tool was developed [7], and was 0.71 in this study.

#### 2.3.3. Problem-Solving Skills

Problem-solving skills (PSS) was measured using the Problem Solving Inventory (PSI) developed by Heppner and Petersen [20], translated by Jeon [21]. The tool, a five-point Likert scale, consisted of 21 items with three sub-areas: confidence, approach-avoidance style, and person control. The higher the score, the higher the PSS. Cronbach’s α was 0.89 when the tool was developed [20], 0.90 in the research of Jeon [21], and 0.86 in this study.

#### 2.3.4. Grit

The Original Grit Scale (Grit-O), which was developed by Duckworth et al. [14] and translated by Lee and Son [22], was used in this study. This five-point Likert scale consists of two sub-areas with twelve items: consistency of interests and perseverance of efforts. Higher scores indicated higher grit. Cronbach’s α was 0.81 when the tool was developed [14], 0.79 in the research of Lee and Son [22], and 0.86 in this study.

#### 2.3.5. Disaster Triage Ability

With the authors’ permission, 20 items were extracted, revised, and supplemented from the Disaster Triage Education in Korea Disaster Life Support—Basic (KDLS-Basic) scale, which was developed by the Central Emergency Medical Center [23]. The content validity was verified by five experts (two emergency medicine specialists, two emergency structural science professors, and one ER specialist nurse) using the Content Validity Index (CVI) with a four-point Likert scale. The questions were adopted after confirming the minimal CVI of all 20 questions (0.99). Correct answers counted for one point, whereas incorrect answers obtained zero points. Higher scores indicated higher DTA, with a perfect score of 20.

### 2.4. Data Collection

During May 2019, following the Institutional Review Board (IRB) approval, we explained the purpose and method of the study to the head of the institution, the nursing department, and emergency unit for approval. Next, consent forms and structured questionnaires were distributed to voluntary participants, who were informed of the study’s purpose and method. Informed consent forms and questionnaires were collected in separate collection boxes and small souvenirs were offered to the participants.

### 2.5. Data Analysis

Data were analyzed with the SPSS/WIN 25.0 program, using descriptive statistics, the independent *t*-test, one-way ANOVA, and Scheffé post-hoc analysis. The correlations between ST, PSS, Grit, and DTA were analyzed using Pearson’s correlation coefficients. The effects of ST, PSS, and Grit on DTA were analyzed using stepwise multiple linear regression analysis.

### 2.6. Ethical Consideration

This study was conducted following approval by the IRB (MJH 2019-04-010-001). To acquire permission for this survey, the author explained the purpose and methods of our study to the head of each institution and the research manager of the nursing department in all participating institutions. The purpose and methods of the study were explained to the voluntary study participants before the informed consent forms and questionnaires were distributed. It was explained to participants that they can withdraw their participation in the study at any time with no disadvantages. To protect their privacy, the study participants were not specified; the consent forms and questionnaire were collected autonomously. In addition, these collections occurred simultaneously. The completed questionnaires were stored in a locked personal drawer and will be discarded immediately upon completion of the study. Furthermore, the computerized data is encrypted and stored in a personal computer accessible only to the primary investigator and will be deleted after three years of storage period.

## 3. Results

### 3.1. General and Severity Classification-Related Characteristics

The mean age of ERTNs was 29.12 ± 4.81, and ‘36+’ showed the highest proportion, at 13.9%. There were 89.2% females, and 85.5% possessed a Bachelor’s degree or less. The mean clinical experience was 70.22 ± 66.33 months, and over half (58.4%) had ‘<5 years’ of experience. The mean experience in the ER was 63.66 ± 56.15 months, and 59.6% had worked ‘<60 months’ in ER. There were 64.5% and 51.8% of participants who had undertaken disaster and disaster triage education, respectively. Finally, 38.6% had severity classification experience, and 60.8% possessed a KTAS qualification (Table 1).

### 3.2. Participants’ Strategic Thinking, Problem-Solving Skills, Grit, and Disaster Triage Ability

The mean ST score was 3.23 ± 0.19 points. When measuring the sub-areas, intelligent opportunism had the highest score (3.53 ± 0.35), followed by futurism (3.26 ± 0.48), conceptual thinking (3.25 ± 0.24), and system thinking (2.86 ± 0.18). The mean PSS score was 3.41 ± 0.39, and by sub-areas, approach-avoidance style 3.60 ± 0.37, confidence 3.47 ± 0.52, and person control 2.83 ± 0.74. The mean grit score was 3.02 ± 0.47, and by sub-areas, perseverance of efforts was 3.13 ± 0.48 and consistency of interests was 2.85 ± 0.60. The mean DTA score was 14.03 ± 4.28 (range, 2–19; Table 2).

### 3.3. Association of Disaster Triage Ability with General and Severity Classification-Related Characteristics

There was no significant difference in DTA according to the general characteristics; however, there was a significant effect of disaster triage education on DTA (t = 2.26, *p* = 0.022); nurses who participated in disaster triage training had significantly higher DTA scores (Table 1).

### 3.4. Association between Strategic Thinking, Problem-Solving Skills, Grit, and Disaster Triage Ability

DTA had a positive correlation with futurism (r = 0.19, *p* = 0.019), a sub-area of STS. Additionally, there was a significant positive and negative correlation with confidence (r = 0.30, *p* < 0.001) and approach-avoidance style (r = −0.28, *p* < 0.001), which are sub-areas of PSI; however, this was only to a weak degree (Table 3).

### 3.5. Factors Influencing Disaster Triage Ability

Multiple regression analysis was conducted to identify the factors influencing DTA in ERTNs. Futurism, confidence, and approach-avoidance style were used as independent variables. Multicollinearity between tolerance limits and independent variables, as well as mutual independence between residuals, were identified. Tolerance was 0.55–0.88, the variance inflation factor (VIF) was <10 (range, 1.14–1.81). This confirmed the low correlation between independent variables without multicollinearity. The Durbin–Watson coefficient was closed to two (range, 1.91–2.01) [20], confirming the independence of the residuals.

The factors influencing DTA were identified as the approach-avoidance style, a sub-area of PSI (β = −0.27, *p* < 0.001); confidence, a sub-area of PSI (β = 0.22, *p* = 0.004); and futurism, a sub-area of STS (β = 0.17, *p* = 0.030). These variables explained 14.1% of the DTA. Among these three factors, approach-avoidance style was the most significant factor. Consequently, the DTA of ERTNs was considered higher when individuals reported lower approach-avoidance styles and higher confidence in PSI, and higher futurism in STS (Table 4).

## 4. Discussion

Interest in disaster medical care has increased and related activities due to the occurrence of various disaster accidents. This demand has strengthened the need for severity classification abilities, which influence the success and failure of response results. ERTNs are ideal medical professionals to make appropriate severity classification choices because of their experiences identifying the severity of casualties according to health issues and resource availability. This descriptive survey study sought to provide a new basis for the educational intervention for improved DTA in ERTNs by identifying the degrees of strategic thinking, problem-solving skills, grit, and disaster triage ability in ERTNs. We found that the mean DTA score of ERTNs was 14.03, which can be converted to over 70.15 points in a 100-point scale. A novel tool was used to measure DTA in this study; therefore, we converted the points calculated to a 100-point scale for comparison and analysis with previous studies. The result of this study was similar to a study with Canadian ERTNs, who had a mean DTA score of 72.2 points [24]. This study used a KTAS based on the Canada Triage and Acuity Scale (CTAS) and ERTNs were assessed using similar classification tools to those in the present study. Interestingly, a previous domestic severity classification study reported different results; DTA was lower in domestic military nursing personnel (63.5 points) [25] and higher in 119 paramedics (75.7 points) [26]. This indicates that nurses who perform different occupational duties show differences in their DTA.

A previous study reported that the mean DTA scores of 119 paramedics, which included nurses, were different according to age, clinical experience, and job title [27]. In contrast, there was no difference in DTA scores associated with general characteristics in this study. However, a direct comparison was difficult, as previous studies did not investigate general characteristics [24] or did not consider their relationship to DTA, although age, clinical experience, and job title have been investigated [28]. A previous study confirmed a significant difference in DTA according to the general characteristics, such as age, clinical experience, and job title of the ERTNs [29]. Taken together, these data suggest that a follow-up study assessing the relationship between DTA and general characteristics is required. These differences are significant for the development of DTA educational programs.

This study confirmed the significant difference in DTA according to the presence of disaster triage education; higher DTA scores were associated with disaster triage education participation. This result is similar to a study on US ERTNs, which showed a significant DTA improvement after a video simulation education course [28], and a study on 119 paramedics and public health care emergency teams, which revealed improvements in timing and DTA after education [30]. According to findings of the National Disaster Health Medical Education [31], opportunities for severity classification education are relatively limited because there are no separately operated severity classification courses, and these skills are only included as a part of disaster-related education. Therefore, specialized disaster triage education courses and programs are required.

Our results revealed that a higher DTA score was associated with enhanced confidence in PSI, higher futurism in STS, and a lower level of the approach-avoidance style. These results were similar to those of a previous study showing that higher confidence and job performance was correlated with a higher DTA score in ERTNs in US general hospitals [28]. Confidence in PSI refers to the ability to choose and apply a solution among various options [20]. It is dependent on basic knowledge and performance frequency [31]. This suggests that education should combine theory and practice. Futurism in STS refers to the future assessment ability and is improved through practice [7]. Approach-avoidance style refers to stress-coping skills, which are dependent on stress defense mechanisms [11]. Research has indicated that enhanced DTA is associated with a well-formed approach-avoidance style, which suggests the need for an approach-avoidance style-forming programs. Furthermore, our results differ from research showing that other emergency medical professionals who use appropriate approach-avoidance style defense mechanisms are healthier in disaster situations [11]. This may be due to a limitation of the appropriate approach-avoidance style defense mechanisms in ERTNs due to constant exposure to emergency situations. Hence, the application of appropriate approach-avoidance style defense mechanism formation education in ERTNs is necessary.

The explanatory power of the factors influencing DTA in ERTNs assessed in this study (approach-avoidance style and confidence in PSI, futurism in STS) was 14.1%. Similarly, previous study results have revealed that confidence in PSI [32] and futurism in STS [33] significantly influence DTA. In contrast to our results, mass-casualty incident simulation training of ERTNs has shown that stresses associated with the approach-avoidance style alone significantly affect DTA [34]. DTA aims to distribute medical resources to casualties with higher probabilities of survival. It is responsible for evaluating patients, communicating with and between professionals; providing initial first aid; allocating medical resources; and monitoring, reassessing and managing the flow of the patient treatment [16]. A study on Swedish ERTNs analyzed the skills and influential factors relating to DTA and confirmed that confidence for PSI [32] and futurism in STS enabled the best choices in regard to future situations [33]. Furthermore, another study assessed mass-casualty incident situation training of nurses, doctors, and other healthcare workers from general hospitals in Thailand. They found that severity classification stress was significantly influenced by individual approach-avoidance style [34], which is in contrast with our study. Taken together, our study and previous data highlight that enhanced confidence in PSI, futurism in STS, and the influence of the approach-avoidance style in PSI can improve DTA.

This study confirmed that the influence of an approach-avoidance style and confidence in PSI, futurism in STS, and disaster triage education affect DTA. The approach-avoidance style in PSI is affected by stress coping skills, and confidence in PSI and futurism in STS are affected by education and practice; therefore, this study has provided data that can be used to develop further strategic thinking education programs.

There are some limitations of this study. We sought participation in a limited area; therefore, generalization is limited. Follow-up studies that use national random data are suggested. Furthermore, repeated research through objective data collection, such as measuring DTA using impartial observers, is suggested because it is difficult to exclude subjective effects with self-report-oriented data collection, as used in this study. Moreover, this study identified approach-avoidance style and confidence on PSI and futurism on STS as significant influencing factors; however, there was a low level of explanatory power. We suggest a follow-up study to elucidate the other influencing factors in DTA.

## 5. Conclusions

This study was conducted in order to provide data on the requirements for the development of DTA in ERTNs who play a major role in severity classification in emergency situations. This was performed by identifying the degree and influential factors on DTA in current ERTNs. Our data revealed at least an intermediate level of DTA in ERTNs, which was significantly increased with disaster triage education. Approach-avoidance style and confidence, sub-areas of PSI, and futurism, a sub area of STS, affected DTA, with an explanatory power of 14.1%.

When developing and applying an educational intervention for DTA, it is important to promote the approach-avoidance style as part of a stress coping program. The preparation and application of stress-coping programs such as mindfulness, meditation, relaxation, and so on, may be considered for ERTNs. Furthermore, the use of learning methods such as conceptual guidance, theory, and task-based learning are crucial. Education increases DTA; therefore, the continuous development and application of disaster triage education programs will be most efficient. It is also necessary to provide an opportunity to maintain and improve nurses’ severity classification ability through repeated learning using virtual reality, augmented reality, and extended reality, and to verify the effectiveness of this approach. Additionally, as a follow-up study, the authors suggest an analysis of patient outcomes and medical costs according to the number of triage experiences and their success or failure. Considering the rapid nurse turnover rate due to the shortage of nursing manpower, we suggest a study to verify the effectiveness of severity classification education according to the period of clinical experience.

## Figures and Tables

**Table 1:** Participants’ general characteristics (*N* = 166).

Characteristics	Categories	N (%)	Mean ± SD	Disaster Triage Ability	
				Mean ± SD	t or F (*p*)
Age (year)	≤30	112(73.5)	29.12 ± 4.81	13.71 ± 4.40	1.70
	31–35	21(12.6)		14.29 ± 4.99	(0.186)
	≥36	23(13.9)		15.48 ± 2.33	
Gender	M	18(10.8)		14.89 ± 3.16	0.90
	F	148(89.2)		13.93 ± 4.40	(0.369)
Education	Bachelor’s or less	142(85.5)		13.89 ± 4.25	−0.99
	Master’s or higher	24(14.5)		14.83 ± 4.49	(0.322)
Total clinical experience (month)	≤59	97(58.4)	70.22 ± 66.33	14.11 ± 3.74	0.19
	60–119	42(25.3)	(Range 12–480)	13.69 ± 5.13	(0.829)
	≥120	27(16.3)		14.26 ± 4.82	
Emergency room experience (month)	≤59	99(59.6)	63.66 ± 56.15	14.18 ± 3.73	0.18
	60–119	42(25.3)	(Range 12–360)	13.71 ± 5.13	(0.837)
	≥120	25(15.1)		13.96 ± 4.89	
Disaster education	Yes	107(64.5)		14.12 ± 4.16	0.33
	No	59(35.5)		13.86 ± 4.53	(0.712)
Disaster triage education	Yes	86(51.8)		14.94 ± 4.13	2.26
	No	80(48.2)		13.13 ± 4.46	(0.022)
Disaster triage-related experience	Yes	64(38.6)		14.14 ± 3.43	0.78
	No	102(61.4)		13.96 ± 4.76	(0.867)
Korean Triage and Acuity Scale (KTAS) certificate	Yes	101(60.8)		14.25 ± 3.91	0.81
	No	65(39.2)		13.69 ± 4.82	(0.417)

**Table 2:** Strategic thinking, problem-solving skills, grit, and disaster triage ability. (*N* = 166).

Variable	Mean ± SD	Min	Max	Range
**Strategic Thinking**	3.23 ± 0.19	2.62	3.92	1–5
Conceptual Thinking	2.86 ± 0.18	2.43	3.43	1–5
System Thinking	3.25 ± 0.24	2.67	3.67	1–5
Futurism	3.26 ± 0.48	1.83	5.00	1–5
Intelligent Opportunism	3.53 ± 0.35	2.71	4.43	1–5
**Problem-Solving Skills**	3.41 ± 0.39	2.67	4.57	1–5
Confidence	3.47 ± 0.52	2.29	4.86	1–5
Approach-avoidance style	3.60 ± 0.37	2.40	4.30	1–5
Person Control	2.83 ± 0.74	1.25	4.75	1–5
**Grit**	3.02 ± 0.47	2.00	4.36	1–5
Consistency of Interests	2.85 ± 0.60	1.50	4.33	1–5
Perseverance of Effort	3.13 ± 0.48	2.00	4.50	1–5
**Disaster Triage Ability**	14.03 ± 4.28	2.00	19.00	0–20

**Table 3:** Correlations among strategic thinking, problem-solving skills, grit, and disaster triage ability (*N*= 166).

r(*p*)	DTA ^1^	ST ^2^					PSS ^7^				Grit		
		Total	CT ^3^	S ^4^	F ^5^	I ^6^	Total	C ^8^	AAS ^9^	PC ^10^	Total	CI ^11^	PE ^12^
**DTA ^1^**	1												
**ST** ^2^	−0.06 (0.452)	1											
CT ^3^	0.01 (0.879)	0.07 (0.376)	1										
S ^4^	−0.02 (0.827)	0.49 (<0.001)	−0.14 (0.078)	1									
F ^5^	0.19 (0.019)	0.80 (<0.001)	−0.05 (0.548)	0.16 (0.035)	1								
I ^6^	0.05 (0.514)	0.73 (<0.001)	−0.24 (0.002)	0.27 (<0.001)	0.36 (<0.001)	1							
**PSS** ^7^	0.03 (0.742)	0.46 (<0.001)	−0.20 (0.008)	0.12 (0.120)	0.60 (<0.001)	0.26 (0.001)	1						
C ^8^	0.30 (<0.001)	0.41 (<0.001)	−0.23 (0.003)	0.15 (0.057)	0.57 (<0.001)	0.19 (0.013)	0.83 (<0.001)	1					
AAS ^9^	−0.28 (<0.001)	0.41 (<0.001)	−0.10 (0.214)	0.11 (0.171)	0.41 (<0.001)	0.32 (<0.001)	0.73 (<0.001)	0.32 (<0.001)	1				
PC ^10^	−0.09 (0.253)	0.26 (0.001)	−0.16 (0.037)	0.02 (0.829)	0.44 (<0.001)	0.07 (0.353)	0.82 (<0.001)	0.64 (<0.001)	0.37 (<0.001)	1			
**Grit**	−0.02 (0.772)	0.32 (<0.001)	−0.15 (0.050)	0.16 (0.045)	0.41 (<0.001)	0.15 (0.058)	0.54 (<0.001)	0.55 (<0.001)	0.24 (0.002)	0.52 (<0.001)	1		
CI ^11^	−0.05 (0.531)	0.14 (0.070)	−0.08 (0.314)	0.14 (0.069)	0.22 (0.004)	−0.02 (0.797)	0.32 (<0.001)	0.39 (<0.001)	0.01 (0.855)	0.37 (<0.001)	0.80 (<0.001)	1	
PE ^12^	0.03 (0.689)	0.32 (<0.001)	−0.22 (0.005)	0.12 (0.139)	0.42 (<0.001)	0.19 (0.014)	0.56 (<0.001)	0.54 (<0.001)	0.32 (<0.001)	0.47 (<0.001)	0.89 (<0.001)	0.53 (<0.001)	1

^1^ Disaster triage ability. ^2^ Strategic thinking. ^3^ Conceptual thinking. ^4^ System thinking. ^5^ Futurism. ^6^ Intelligent opportunism. ^7^ Problem-solving skills. ^8^ Confidence. ^9^ Approach-avoidance style. ^10^ Person control. ^11^ Consistency of interests. ^12^ Perseverance of efforts.

**Table 4:** Influencing factors on disaster triage ability among emergency room nurses (*N* = 166).

Variables		Disaster Triage Ability					
		B	β	*t*	*p*	Tolerance	VIF ^1^
(constant)		12.00		2.88	0.005		
Problem-Solving Skills	Approach-avoidance style	−3.67	−0.27	−3.22	<0.001	0.88	1.14
	Confidence	2.16	0.22	2.16	0.004	0.58	1.73
Strategic Thinking	Futurism	1.20	0.17	1.55	0.030	0.55	1.81
		R^2^ = 0.150, Adjusted R^2^ = 0.141, F = 8.91, *p* < 0.001					

^1^ VIF: variance inflation factor.

## Data Availability

The data presented in this study are not publicly available due to participants’ privacy.
